# Empirical models for compressive and tensile strength of basalt fiber reinforced concrete

**DOI:** 10.1038/s41598-023-47330-2

**Published:** 2023-11-14

**Authors:** Muhammad Asghar, Muhammad Faisal Javed, M. Ijaz Khan, Sherzod Abdullaev, Fuad A. Awwad, Emad A. A. Ismail

**Affiliations:** 1 Department of Geotechnical Engineering, NICE, National University of Science and Technology, Islamabad, Pakistan; 2 Department of Civil Engineering, COMSATS University Islamabad, Abbottabad Campus, Islamabad, Pakistan; 3 Department of Mechanical Engineering, Lebanese American University, Beirut, Lebanon; 4 Department of Mathematics and Statistics, Riphah International University I-14, Islamabad, 44000 Pakistan; 5 Department of Mechanics and Engineering Science, Peking University, Beijing 100871, China; 6 Faculty of Chemical Engineering, New Uzbekistan University, Tashkent, Uzbekistan; 7 Department of Science and Innovation, Tashkent State Pedagogical University Named After Nizami, Bunyodkor Street 27, Tashkent, Uzbekistan; 8 Department of Quantitative Analysis, College of Business Administration, King Saud University, P.O. Box 71115, 11587 Riyadh, Saudi Arabia

**Keywords:** Engineering, Civil engineering

## Abstract

When molten magma solidifies, basalt fiber (BF) is produced as a byproduct. Due to its remaining pollutants that could affect the environment, it is regarded as a waste product. To determine the compressive strength (CS) and tensile strength (TS) of basalt fiber reinforced concrete (BFRC), this study will develop empirical models using gene expression programming (GEP), Artificial Neural Network (ANN) and Extreme Gradient Boosting (XG Boost). A thorough search of the literature was done to compile a variety of information on the CS and TS of BFRC. 153 CS findings and 127 TS outcomes were included in the review. The water-to-cement, BF, fiber length (FL), and coarse aggregates ratios were the influential characteristics found. The outcomes showed that GEP can accurately forecast the CS and TS of BFRC as compared to ANN and XG Boost. Efficiency of GEP was validated by comparing Regression (R^2^) value of all three models. It was shown that the CS and TS of BFRC increased initially up to a certain limit and then started decreasing as the BF % and FL increased. The ideal BF content for industrial-scale BF reinforcement of concrete was investigated in this study which could be an economical solution for production of BFRC on industrial scale.

## Introduction

Basalt fibers (BF) are produced from the basalt rocks which are formed because of volcanic lava solidification. BF are abundantly found on earth due to their volcanic origin^1,2^. BF have higher viscosity and resistivity of temperature shifts from low to high which ascribes higher Compressive Strength (CS) and durability^1^. Micrograph Analysis show that BF have good adhesion properties which result in durable and high strength structures. Application of BF as a substitute for glass fibers (GF) has grown quickly over the past 2 decades^3^. Nearly no change was found in Young’s Modulus for BF after exposure in different types of media. Road infrastructure, pipes, bridge components, transportation-related projects, sporting goods, wind turbines, offShore structures, etc. all employ BF^3,4^. From above reviews it can be observed that BF have been used in many scientific investigations including alkali resistance of BF and their influence on asphalt mixtures on different temperatures, however, further study on enhancing CS and Tensile Strength (TS) of BF needs improvement^5,6^.

Properties of concrete had been found considerably fluctuating with changes in cement matrix. Based on statistical data, mix design of concrete and specially the type of material used as an additive in concrete effects the properties of concrete^7^. By substituting alternative fibers for a portion of the cement, fiber reinforced concrete's impact resistance can be improved to a great extent^8^. The ultra-high performance of concrete can be achieved by fiber reinforcement technique and can be further increased with hybridization of fibers^9^. Similarly, Nylon fibers are also used for reinforcement of concrete, but they can immerse in the mixing water, and as a consequence, decrease the workability. If microfibers are used the use of Nylon Fibers at a minute fiber volume is restricted due to these characteristics. In addition, Nylon fibers are expensive as compared to other fibers. Comparative studies have shown more desirable results with reinforcement of BF^10^.

Not only the outcome is more precise but there is enhancement in physio-mechanical properties and ductile behavior. There is a slight decrease of 2% in volume of BFRC due to volumetric strain instability while during reinforcement of BF^11^. The first use of BF was reported in 1998 in a report published by Highway Innovations Deserving Exploratory Analysis (IDEA) Project 45 working ability of 3-D BFRC and Basalt Rod reinforced concrete were investigated. BFRC concrete impart high energy absorption ability and improvise ductility^12^. BF are preferred over steel and poly vinyl alcohol (PVA) fibers because they show efficient thixotropic behavior, respectively. BFRC when used in pavement and structural element shows more satisfactory results with 90% load transfer in the member. When the water-to-cement (w/c) ratio is kept constant at 0.24, the CS of concrete containing BF minimizes due to high quantity of fiber in concrete. After 2 days, the biggest reduction in CS—from 15.5 to 18.2%—occurs when the fiber concentration rises from 2 to 10 kg/m^3^. After 7 and 28 days of curing, a comparable performance can be assessed. BF with a length of 24 mm performed better than BF with a length of 12 mm in terms of CS^13^. This can be attributed to the fact that a longer anchoring period makes it possible to hold mortar particles more firmly. At the same time, the longer BF has a dominant and significant impact on the CS as well as TS of the mortar specimen. BF have shown their ability to upgrade the mechanical properties of concrete^14^. The average CS has increased by 11% with BFRC. Depending on the longitudinal reinforcement ratio, increasing the fiber volume fraction also increased the BFRC slab strips' ability to support more weight. Several experiments had shown a considerable increase in CS, anti-dry shrinkage cracking resistance and modulus of rupture in BFRC^15^. The damage fatigue patterns at higher and lower stress levels can be overcome by using BFRC. With multiscale numeric simulation of BFRC, it has been seen that at low fiber length and content (below 12 mm and 2%) CS of BFRC had found to be increasing^16^.

To achieve effective model of BFRC having higher CS, more operative equations should be generated which can be done through Artificial Neural Networks (ANNs) using Gene Expression Programming^17,18^. ANNs are a useful tool for assessing concrete's performance. It has been demonstrated using ANNs that concrete with fiber reinforcement produces concrete that is highly durable and cost-effective^19,20^. According to experimental data, the concrete CS classifier utilising ANNs performs better than the previously recommended techniques^21,22^. Further research is mandatory for adding more input attributes and using a smaller number of iterations. Prediction of different proportions of concrete give better results and consume less time using ANNs^23,24^. When ANNs are used to estimate the CS of concrete, the results are accurate and have good mix design. However, minimum errors have been seen with large size dataset and correct prediction rate needs more improvement^25–27^. Numerous studies found that the intricacy of the created ANN models caused them to be overfitted in contrast to design code values. Additionally, multi-collinearity proved a problem in this criterion^28–30^. Modified ANN techniques are also used to check CS of concrete. ANN may prove as an advanced predictive tool but several tools working on ANNs like Gene expression programming (GEP) yield more suitable and desired results.

GEP is one of the methods of artificial neural networks and is highly efficient with distinct functions. It allows algorithms to execute the results more effectively thus greatly surpassing the other functions. Since input or people are encoded as linear strings of set length before being expressed as non-linear units of unusual sizes and shapes (genomes), GEP use is preferred (expression trees or simple diagram representation). The strong R^2^ values prove the applicability of GEP models for forecasting the compressive and TS of concrete. Another benefit of using GEP is its ability to combine multiple simple programs into a single complex program^31^. Furthermore, using GEP for parametric analysis supplies a greater knowledge for evaluating performance of different mix-proportions of concrete. Hence, several research studies proved that GEP is most precise and efficient form of traditional genetic programming techniques. Till now, no precise and accurate models are available on CS and TS of BFRC. In this research, CS and TS of BFRC have been modelled based on most effective parameters using GEP.

This study provides the effectiveness of different parameters in the production of BFRC. It focuses on the development of efficient empirical equations to predict the compressive and tensile strength of BFRC without compromising on the accuracy provided by ANN and other techniques. It focuses on giving optimum content of BF of different lengths and different parameters in BFRC by utilizing the same equation. In addition, SHAP analysis is conducted to check the effect of different parameters on the compressive and tensile strength of BFRC. Moreover, this research provides the accuracy of GEP in predicting strengths (CS and TS) of BFRC.

## Research methodology

The methods used to produce the empirical model for CS and TS of BFRC are described in this section. GEP will be addressed in detail, and then the discussion of the method used in this study will follow.

### Overview of gene expression programming

GEP models are more functional and yield correct results by using the optimized parameters. GEP is more advanced and expanded form of gene programming (GP), a type of machine learning that generates models which rely on genetic evaluation^31,32^. GEP is a new technique for the advancement of computer programs that relies on the character linear chromosomes constituting of genes structurally organized in a head and a tail. As a result of mutation, transposition, root transposition, gene transposition, gene recombination, and one- and two-point recombination, the chromosomes play the function of genomes that are subject to alteration. The targets to be chosen are expressed trees, which are encoded by the chromosomes^33–35^. The method can run with high efficiency, which is significantly better than current adaptive algorithms, thanks to the development of these different entities (genome and expression trees) with discrete purposes. Genetic programming's constant linear width makes GEP an efficient method^36^. The simplest criteria used in GEP also allow for the development of complicated and non-linear programs due to multi-genic behavior because of the genetic process occurring at the chromosomal level. Five sets made up the entire GEP^37^: the Function set, the Terminal set, the Fitness measure set, the Parameters set, and the Criteria set. In GEP, each specimen is set as a genome, which is a fixed-size linear string. Furthermore, during the reproduction stage, the genetic operators are used for modification of chromosomes.

The process goes ahead by initially selecting data randomly, then the best combination of population is selected based on error criteria and outliers are separated. Further, the most suitable combination is produced by mutation, crossover and direct cross-over^38^. This process is also named as “learning”. After running several cycles most suitable model is created by reaching maximum iterations. GEP models are less time consuming and more efficient than early used traditional experimental procedures for predicting strength of concrete composites.

The summary of the processes undergoing GEP modelling continue in the following manner.

1. Based on recorded data (population) number of chromosomes are produced randomly.
2. The chromosomes formed in the first step then generate mathematical equations.
3. Each chromosome is then used to check suitability with targeted function. This is an iterative process and if iterations do not stop then best of the first generation is selected using roulette wheel method.
4. To create modified individuals from other chromosomes the genetic operators are applied which are the GEP algorithm.
5. Now more chromosomes are created by several iterations for a certain number of generations and the model is formed with most efficient producing results.

### Artificial neural networks (ANNs)

Artificial neural networks (ANNs) are a class of machine learning algorithms inspired by the structure and functioning of biological neural networks, such as the human brain^1^. ANNs consist of interconnected nodes, or artificial neurons, organized into layers: input layer, hidden layers (if any), and an output layer. These networks are used for a wide range of tasks, including pattern recognition, regression, classification, and more. Here's an overview of ANNs and their potential applications in civil engineering:

Basic Structure of Artificial Neural Networks:

- *Inner Layer* Receives the raw data or features for the task.
- *Hidden Layers* One or more layers of neurons that process and transform the input data.
- *Output Layer* Produces the final output, which could be a prediction, classification, or any other relevant result.

ANNs are trained using labeled data, where the correct outputs are known.

During training, the network adjusts its internal parameters (weights and biases) to minimize the error between predicted and actual outputs. Popular optimization algorithms like backpropagation are used for this purpose. ANNs can analyze sensor data from bridges, buildings, or other infrastructure to detect signs of damage or wear and tear. They can predict the remaining useful life of structural components, helping with maintenance planning^2^. ANNs can model soil behavior, helping predict settlement, slope stability, and bearing capacity. They can analyze geological data to identify potential hazards like landslides or earthquakes. ANNs can predict traffic flow, congestion patterns, and optimize traffic signal timings. They can be used for predictive maintenance of road infrastructure based on traffic data. ANNs can model complex hydrological systems to predict river flow, rainfall, and flood events. They can assist in flood risk assessment and early warning systems^3^. ANNs can help optimize construction schedules and resource allocation. They can predict construction project delays and cost overruns based on historical data. ANNs can analyze data from material testing to predict material properties and behavior under various conditions. ANNs can model the environmental impact of civil engineering projects and help with mitigation strategies. ANNs can assist in urban planning by predicting population growth, traffic patterns, and land-use changes. ANNs can be used to monitor the quality of construction materials, such as concrete or asphalt, based on inspection data. In all these applications^4^. ANNs excel at handling complex, nonlinear relationships in the data, which are often challenging to capture with traditional engineering models. However, it's essential to have enough high-quality data for training and to validate the ANN's performance for reliable results in civil engineering applications^5^.

### Extreme gradient boost (XG Boost)

Extreme gradient boosting (XG Boost) is a powerful and popular machine learning algorithm used for both classification and regression tasks. It belongs to the gradient boosting family of algorithms and is known for its high performance, efficiency, and versatility. XG Boost has gained popularity in various fields, including finance, healthcare, and natural language processing^6^. While it may not be a common tool in traditional civil engineering, it can still be applied to certain civil engineering problems. XG Boost is an ensemble learning algorithm that combines the predictions of multiple decision trees to create a robust and accurate model.

It is called "Extreme" Gradient Boosting because it emphasizes the use of gradient boosting techniques, which iteratively optimize the model's performance. XG Boost is highly efficient and parallelizable, making it suitable for large datasets and distributed computing environments. Civil engineering structures, such as bridges and roads, require regular maintenance to ensure their safety and longevity^7^. XG Boost can be used to predict maintenance needs by analyzing data related to factors like structural wear and environmental conditions. In construction projects, XG Boost can help identify defects and quality issues by analyzing data from sensors, cameras, or other monitoring equipment. It can flag anomalies and potential problems in real-time, enabling timely interventions^8^.

XG Boost can be used to forecast traffic patterns, optimize traffic signal timings, and even predict congestion or accidents. Civil engineers can utilize these predictions to plan better transportation systems. XG Boost can be used to model and predict the environmental impact of construction projects, aiding in decision-making and regulatory compliance^9^. XG Boost can help analyze soil properties and geological data for construction site suitability and stability assessments. It can predict factors like soil settlement, groundwater levels, or landslides, which are crucial for safe construction. XG Boost can assist in estimating project costs by analyzing historical data, construction methods, and market conditions. Accurate cost estimates are vital for project planning and budgeting.

Applying machine learning in civil engineering requires domain knowledge and high-quality data. Ensuring the data used in XG Boost models is accurate and representative is critical. Model interpretability is important, especially in engineering fields, to understand why certain predictions are made. XG Boost is a versatile machine learning algorithm that can be applied to various aspects of civil engineering, from predictive maintenance to environmental impact assessment and traffic management. It has the potential to enhance decision-making, improve safety, and optimize infrastructure projects in this field, if domain expertise and high-quality data are applied appropriately^10^.

### Data collection

The study of past research led to the collection of data on the CS of BFRC. It should be noted that numerous tests were conducted to figure out the database's veracity^11–35^

The study of past research led to the collection of data on the CS of BFRC. This produced 153 datasets for CS (*CS*) and 127 datasets for TS (*TS*), which were used to create the corresponding empirical models. The training, validation, and testing sets of the database were randomly chosen for this investigation. The model was trained using the training data, and the validation data was used to confirm the model's generalizability. Throughout the testing process, many expressions were assessed on the collected data.

In Table 1, the descriptive statistics are displayed. It is recommended to employ the provided formulas for this set of data to make accurate forecasts of the CS and TS.

**Table 1:** Descriptive statistics of input parameters.

Parameter	w/c	Coarse aggregate	Fine aggregate	Fiber length	CS	TS
Mean	0.49	1038.60	668.77	17.38	49.15	4.1035
Standard error	0.01	17.24	10.45	0.67	1.84	0.35
Median	0.5	1052	680	18	42.32	3.52
Mode	0.55	1400	770	12	37.4	3.39
Standard deviation	0.11	213.26	129.31	8.39	22.84	1.58
Sample variance	0.01	45,482.45	16,721.11	70.49	521.74	2.49
Kurtosis	0.33	− 0.35	− 0.20	2.73	1.70	1.53
Skewness	− 0.41	− 0.22	0.47	1.25	1.37	1.59
Range	0.58	800	496	44	106.3	5.38
Minimum	0.22	600	446	6	17	2.42
Maximum	0.8	1400	942	50	123.3	7.8
Sum	75.23	158,907.32	102,323	2659.9	7520.88	3877.94
Count	153	153	153	153	153	127

It should be noted that numerous tests were conducted to evaluate the database's consistency and validity. The datasets that considerably (up to 20%) diverged from the overall trend were regarded as negligible while developing or assessing the performance of the models.

**Figure 1:**
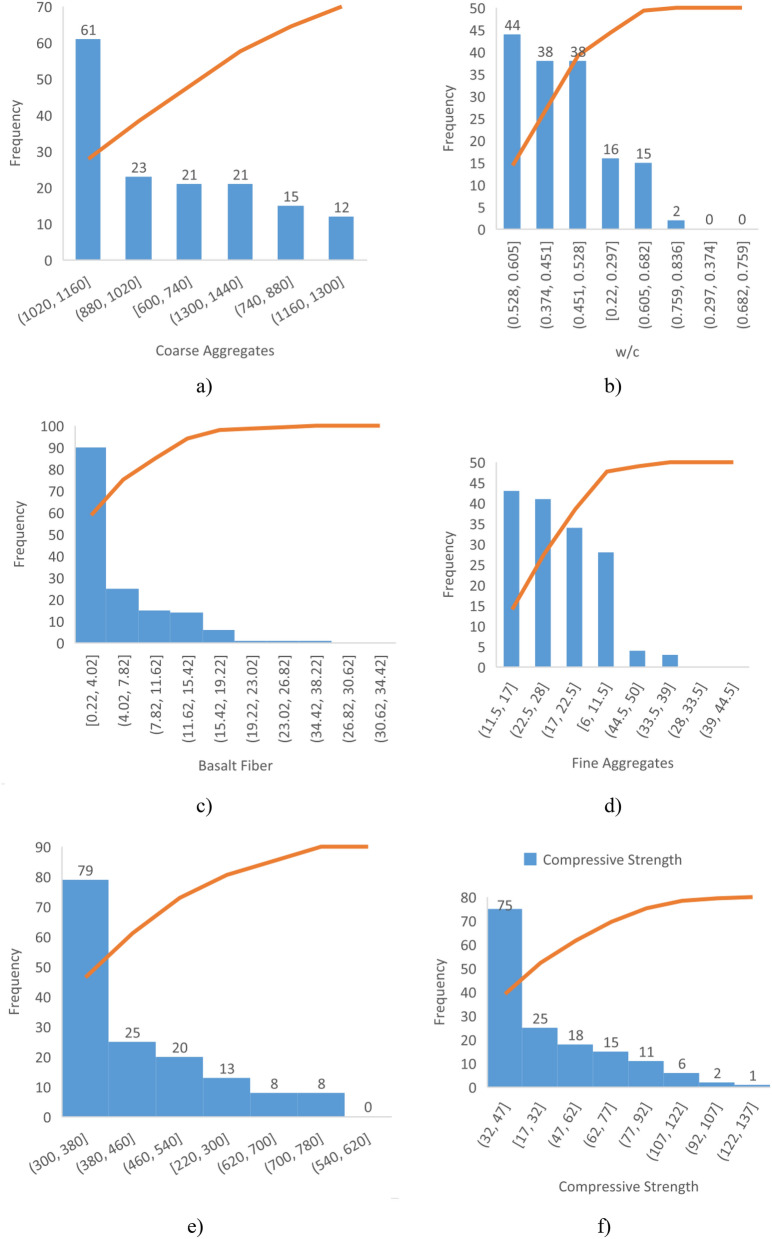
Contribution of (**a**) CA, (**b**) w/c, (**c**) BF, (**d**) FA, (**e**) Cement and (**f**) CS of BFRC.

**Figure 2:**
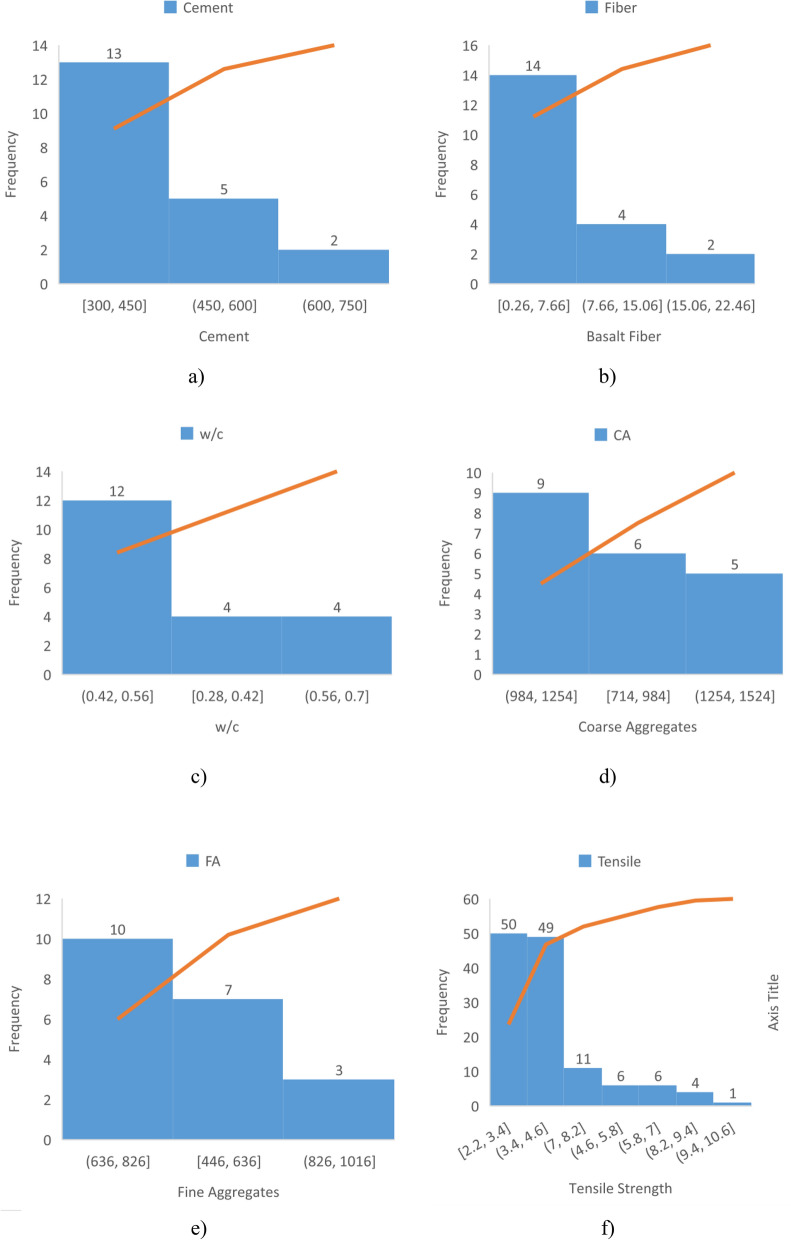
Contribution of (**a**) cement, (**b**) BF (**c**) w/c (**d**) CA (**e**) FA and (**f**) TS of BFRC.

The contribution of different input parameters for design of CS and TS of BFRC can be seen in Figs. 1 and 2. These input parameters played a part in the assessment of optimum CS and TS of BFRC.

### Model development

Prior to developing the model, the first step is to decide which input parameters will have an impact on the BFRC's parameters. To assess the most influential parameters on the characteristics of the BFRC and establish a generalized connection, all the concerned factors in the studied data were carefully analyzed, and the efficiency of multiple preliminary runs was recorded. As a result, it is believed that the variables in Eq. 1 are functions of the CS of BFRC. The robustness and generalizability of the resulting model depend heavily on the fitting parameters, it should be noted. Based on recommendations from the literature and numerous test runs, the fitting parameters for the GEP method were selected.

1 $$CS\;and\;TS = f\left( {w/c, \, CA, \, FA, \, BF, \, cement} \right)$$

How long the module will run for is decided by the size of population (number of chromosomes). Depending on the intricacy of the prediction model, 50, 100, or 150 levels were used as the population size. The architecture of many models created by software is determined by the head size and gene count, where the former considers the intricacy of every term and the latter the number of sub-ETs in the model. Three head sizes—5, 8, or 10—and a set number of 3 and 5 genes were used in this experiment. A list of the precise parameters used in the GEP algorithm for the two models can be found in Tables 2 and 3.

**Table 2:** Summary of best GEP models for CS of BFRC.

Trial no	No. of inputs, used variables	No. of chromosomes	Head size, number of genes	Program size, no of literals	Duration (min)	Training dataset			Linking function
CS model						R^2^	RMSE	R	
Parameters for running the program									
GEP 1^a,m^	6.4	30	8.3	43.16	120	0.87	8.87	0.93	Addition
GEP 2^a,m^		30	8.3	39.10	100	0.88	8.71	0.94	Addition
GEP 3^a,m^	6.5			44.17	80	0.93	6.48	0.96	Addition
GEP 4^b,m^		50	8.3	37.12	70	0.84	9.91	0.92	Addition
GEP 5^c,n^				42.12	60	0.85	9.57	0.92	Addition
GEP 6^d,m^	6.6	50	10.5	83.24	60	0.91	7.49	0.95	Addition
GEP 7^a,m^				81.26	50	0.89	7.88	0.94	Addition
GEP 8^a,m^				77.26	40	0.90	7.74	0.95	Multiplication
GEP 9^e,p^		100	10.3	49.13	120	0.81	8.88	0.91	Addition
GEP 10^b,m^				49.18	100	0.82	10.49	0.90	Addition
GEP 11^f^^,m^				49.15	80	0.91	7.22	0.96	Addition
GEP 12^g^^,m^		150	8.5	65.23	70	0.85	9.58	0.98	Addition
GEP 13^c,m^				68.18	60	0.88	8.45	0.94	Subtraction
GEP 14^h^^,m^				56.14	50	0.93	6.53	0.96	Addition
GEP 15^b,m^				72.19	40	0.88	8.46	0.94	Addition
GEP 16^i,n^	5.4	200	3.5	28.8	120	0.84	9.82	0.92	Addition
GEP 17^j,o^				33.10	100	0.85	9.44	0.93	Addition
GEP 18^k^^,p^			5.5	42.13	80	0.86	9.36	0.92	Addition
GEP 19^g^^,m^				40.10	70	0.89	8.08	0.94	Addition
GEP 20^l^^,p^				37.9	60	0.91	9.58	0.85	Addition

^a^The operations employed included +, −, *, /, sqrt, x^3^.

^b^The operations employed included +, −, *, /, sqrt, x^3^, x^2^.

^c^The operations employed included +, -, *, /, sqrt, x^3^, x^2^, 3Rt.

^d^The operations employed included +, −, *, /, sqrt, x^3^, exp, sin, cos, atan, ln.

^e^The operations employed included +, −, *, /, sqrt, x^3^, x^2^, 3Rt.

^f^The operations employed included +, −, *, /, sqrt, x^3^, x^2^, pow.

^g^The operations employed included +, −, *, /, sqrt, x^3^, exp, sin, cos.

^h^The operations employed included +, −, *, /, sqrt, x^3^, x^2^,3Rt,4Rt, exp, ln.

^i^The operations employed included +, −, *, /, sqrt.

^j^ The operations employed included +, −, *, /, sqrt, x^3^, exp, sin, cos, atan,ln.

^k^The operations employed included +, −, *, /, sqrt, x^3^, x^2^.

^l^The operations employed included +, −, *, /, sqrt, x^3^, x^2^,3Rt,4Rt, exp, ln.

^m^The weight of the “+, −, *” operations was four times that of others.

^n^The weight of the “+, −, *” operations was seven times that of others.

^o^The weight of the “*” operations was four times that of others.

^p^The weight of the “+, −, *” operations was three times that of others.

**Table 3:** Selection of best GEP model for TS of BFRC.

Trial no	No. of inputs used variables	No. of chromosomes	Head size, number of genes	Program size, no of literals	Duration (min)	Training Dataset			Linking function
TS model						R^2^	RMSE	R	
Parameters for running the program									
GEP 1^a,m^	6.4	30	8.3	36.9	120	0.81	0.64	0.89	Addition
GEP 2^a,m^		30	8.3	39.9	100	0.78	0.68	0.88	Addition
GEP 3^a,m^	6.5			42.10	80	0.72	0.77	0.85	Addition
GEP 4^b,m^		50	8.3	36.7	70	0.75	0.73	0.85	Addition
GEP 5^c,n^				36.9	60	0.77	0.70	0.87	Addition
GEP 6^d,n^	6.6	50	10.5	79.23	60	0.81	0.64	0.90	Addition
GEP 7^a,m^				77.22	50	0.83	0.59	0.91	Addition
GEP 8^a,m^				79.27	40	0.85	0.56	0.92	Multiplication
GEP 9^e,p^		100	10.3	33.11	120	0.87	0.73	0.87	Addition
GEP10^b,m^				49.18	100	0.89	0.75	0.87	Addition
GEP11^f^^,m^				49.15	80	0.91	0.72	0.96	Addition
GEP12^g,m^		150	8.5	64.16	70	0.83	0.60	0.91	Addition
GEP13^c,m^				76.18	60	0.85	0.72	0.94	Subtraction
GEP14^h,m^				78.14	50	0.83	0.71	0.94	Addition
GEP15^b,m^				78.19	40	0.87	0.71	0.94	Addition
GEP16^i,n^	5.4	200	3.5	32.6	120	0.88	0.68	0.88	Addition
GEP17^j,o^				38.11	100	0.86	0.59	0.87	Addition
GEP18^k,p^			5.5	42.10	80	0.85	0.66	0.92	Addition
GEP19^g,m^				40.8	70	0.84	0.88	0.94	Addition
GEP20^l,p^				35.6	60	0.84	0.85	0.85	Addition

^a^The operations employed included +, −, *, /, sqrt, x^3^.

^b^The operations employed included +, −, *, /, sqrt, x^3^, x^2^.

^c^The operations employed included +, −, *, /, sqrt, x^3^, x^2^, 3Rt.

^d^The operations employed included +, −, *, /, sqrt, x^3^, exp, sin, cos, atan, ln.

^e^The operations employed included +, −, *, /, sqrt, x^3^, x^2^, 3Rt.

^f^The operations employed included +, −, *, /, sqrt, x^3^, x^2^, pow.

^g^The operations employed included +, −, *, /, sqrt, x^3^, exp, sin, cos.

^h^The operations employed included +, −, *, /, sqrt, x^3^, x^2^,3Rt,4Rt, exp, ln.

^i^The operations employed included +, −, *, /, sqrt.

^j^The operations employed included +, −, *, /, sqrt, x^3^, exp, sin, cos, atan, ln.

^k^The operations employed included +, −, *, /, sqrt, x^3^, x^2^.

^l^The operations employed included +, −, *, /, sqrt, x^3^, x^2^,3Rt,4Rt, exp, ln.

^m^The weight of the “+, −, *” operations was four times that of others.

^n^The weight of the “+, −, *” operations was seven times that of others.

^o^The weight of the “*” operations was four times that of others.

^p^The weight of the “+, −, *” operations was three times that of others.

Correlation coefficient is an often employed performance indicator (R). R, however, cannot be used as the primary sign of the model's prediction accuracy because it is insensitive to the division and multiplication of output numbers. Root means square error (RMSE) and the R^2^ regression component are therefore also considered in this study. The model's performance is assessed using a performance index (β), which functions as a function of the RMSE, R^2^, and R. These error functions of mathematical expressions are provided in Eqs. (2)–(7).

2 $${\text{RMSE }} = \sqrt {\frac{{\mathop \sum \nolimits_{i = 1}^{n} \left( {e_{i} - m_{i} } \right)\left( {e_{i} - m_{i} } \right)}}{n}}$$

3 $${\text{MAE }} = \frac{{\mathop \sum \nolimits_{i = 1}^{n} \left| {e_{i} - m_{i} } \right|}}{n}$$

4 $${\text{RSE }} = \frac{{\mathop \sum \nolimits_{i = 1}^{n} \left( {m_{i} - e_{i} } \right)\left( {m_{i} - e_{i} } \right)}}{n}$$

5 $${\text{RRMSE }} = \frac{1}{{\left| {\overline{e}} \right|}}\sqrt {\frac{{\mathop \sum \nolimits_{i = 1}^{n} (\left( {e_{i} - m_{i} } \right)^{2} }}{n}}$$

6 $${\text{R }} = \frac{{\mathop \sum \nolimits_{i = 1}^{n} \left( {e_{i} - \overline{{e_{i} }} } \right)\left( {m_{i} - \overline{{m_{i} }} } \right)}}{{\sqrt {\mathop \sum \nolimits_{i = 1}^{n} \left( {e_{i} - \overline{{e_{i} }} } \right)^{2} } \mathop \sum \nolimits_{i = 1}^{n} \left( {m - \overline{{m_{i} }} } \right)^{2} }}$$

7 $$\beta \, = \frac{RRMSE}{{1 + R}}$$

where *e*_*i*_ and *m*_*i*_ are the *i*th experimental and model outputs, respectively; *e*¯_*i*_ and *m*¯_*i*_ denote the average values of the experimental and model outputs, respectively and *n* stands for the total number of samples. A better model calibration is shown by a high R and a low RMSE value. R values more than 0.8 (1 for an ideal fit) are thought to prove a strong correlation between expected and measured values. An objective function with a lower value (closer to zero) shows efficient model performance. The value of the objective function spans from 0 to positive infinity.

Overfitting of models because of extensive data training is a concern in machine learning. The testing error may extensively while the training mistakes may continue to reduce. To avoid the model overfitting, the optimal model is chosen by optimizing an objective function (OBF), which is represented as under. The fitness function is defined as OBF here.

8 $${\text{OBF }} = \left( {\frac{{n_{T} - n_{v} }}{n}} \right)\beta_{T} + 2\left( {\frac{{n_{v} }}{n}} \right)\beta_{v}$$

where the subscripts T and V define training and validation (or testing) data, respectively, and n depicts the total number of data points. The relative percentages of the training and validation sets of dataset entries are shown by OBF together with the R and RMSE effects. As a result, the minimization of OBF can be considered a precise indicator of the models' overall effectiveness. The ideal model is represented by a value close to zero. Keeping in mind that after simulating 18 different fitting parameter combinations, the model with the lowest OBF was selected.

The implementation of Artificial Intelligence (AI) techniques have demerited the problem of collinearity and the value of coefficient correlation is with in the prescribed limits which can be viewed from Tables 4 and 5. The problem of interdependency among input variables is a common problem which arises during modelling. Efficiency of the developed model reduces due to rise in the strength of model under consideration. To overcome this issue, Correlation Coefficient R is calculated which should have a value less than 0.8. It can be easily viewed from the Tables 4 and 5 that R value is less than 0.8 and hence there is no risk of multi-collinearity among input parameters during modelling.

**Table 4:** Correlation coefficient among input variables used in modelling of CS.

CS model	Cement	Fiber	w/c	Coarse aggregate	Fine aggregate	Fiber length	Experimental strength
Cement	1						
Fiber	−0.20	1					
w/c	−0.5	−0.07	1				
Coarse aggregate	−0.45	0.17	0.12	1			
Fine aggregate	−0.17	−0.04	0.62	0.34	1		
Fiber length	−0.28	0.20	0.29	−0.09	−0.01	1	
Experimental strength	0.56	0.03	−0.80	−0.33	−0.76	−0.16	1

**Table 5:** Correlation coefficient among input variables used in modelling of TS.

TS model	Cement	Fiber	w/c	Coarse aggregate	Fine aggregate	Fiber length	Experimental strength
Cement	1						
Fiber	−0.01	1					
w/c	−0.24	−0.22	1				
Coarse aggregate	−0.36	0.10	−0.05	1			
Fine aggregate	−0.04	−0.09	0.65	0.31	1		
Fiber length	−0.16	0.02	0.26	−0.19	−0.05	1	
Experimental strength	0.34	0.28	−0.51	−0.53	−0.71	0.09	1

## Results and discussion

**Figure 3:**
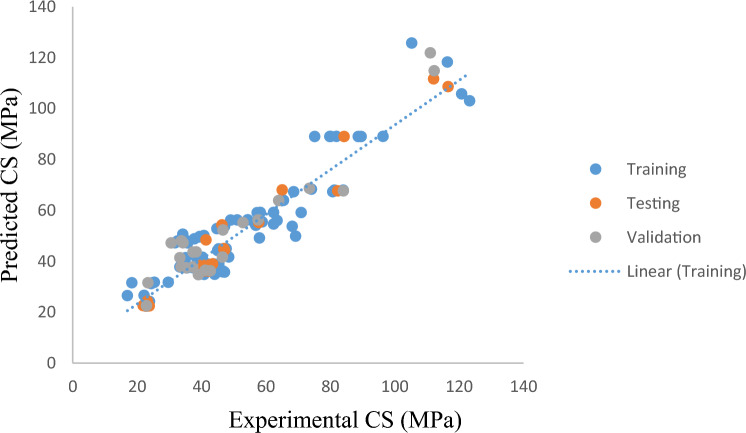
Comparison of CS of BFRC with model prediction.

As previously noted in Tables 2 and 3, the empirical relationships for CS and TS are determined using the four fundamental mathematical operations + ,, x, and. A visual comparison between the projected model and the data for CS is shown in Fig. 3. As shown in Fig. 3, the constructed model clearly considers the effects of each CS and BFRC input parameter. The outcomes displayed in Fig. 3 demonstrate statistical significance; the training, testing, and validation data points nicely fit the trend line, demonstrating the accuracy of the data, accordingly. The number of datasets has a significant impact on the suggested model's reliability. 153 samples of CS were gathered for this study from various sources to help improve outcomes.

### Formula development of *CS* of BFRC

The model for formulating *CS* is created by choosing 3, 5 and 10 for the number of genes and head size, respectively, as shown in Table 2. The 28-day *CS* of the BFRC up to 123 MPa is proposed to be predicted using the simplified equation, Eqs. (9–12). The highlighted value is considered as most suitable for the formulation of *CS*.

9 $$CS\left( {{\text{MPa}}} \right) = Strength = A \times B \times C$$

where

10 $$A = Cement - \left( {fiber \% } \right) - \left( {fiber length} \right) + \left( {214 \div \frac{w}{c}} \right)$$

11 $$B = \frac{6.74}{{FA - FL - 229}}$$

12 $$C = 5.7 - \log \left( {25.25 \times \frac{cement}{{CA}}} \right)$$

### Formula development of *TS* of BFRC

The model for formulating *TS* is made by selecting 3, 5 and 10 for the number of genes and head size, respectively, as shown in Table 3. The 28-day *TS* of the BFRC up to 7.99 MPa is proposed to be predicted using the simplified expression, Eqs. (13–17). The value that has been shown is thought to be ideal for the formation of *TS*.

13 $$TS\left( {{\text{MPa}}} \right) = a \times b \times c \times d$$

where

14 $${\text{a }} = \frac{1}{{8.88FA - CA^{2} - \left( {cement - 10.96} \right)\left( {FA \times Cement} \right) }}$$

15 $${\text{b }} = {\text{ log}}\left( {\frac{Fiber}{{water \times CA^{2} }}} \right) - \frac{cement}{{4.12}}$$

16 $${\text{c }} = {\text{ FA }}{-} \, \left( {\left( {{\text{cement }} + { 846}.{19}} \right) \, \times \, \left( {{\text{cement}}} \right)} \right) \, \times \, \left( {{\text{Fiber }} + \left( {\frac{FA \times CA}{{4.97}}} \right)} \right)$$

17 $${\text{d }} = {\text{ log }}({1}.{66}\left( {{\text{cement }} \times {\text{ Fiber}}} \right) \, {-} \, \left( {{4}.{68 } \times {\text{ Fiber Length}}} \right) \, + { 78}.{48}$$

**Figure 4:**
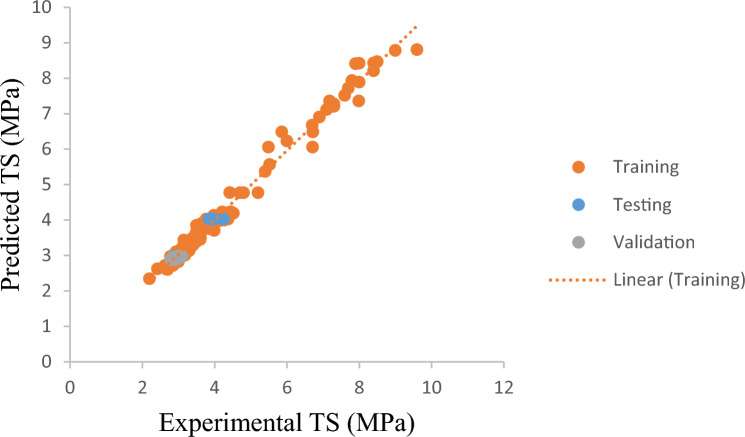
Comparison of TS of BFRC with predicted model.

Figure 4 provides a visual representation of the comparison between the model predictions and the actual results for *TS*. The developed model clearly considers the effects of all *TS* of BFRC input parameters, as can be shown in Fig. 4. Regression lines' slopes for training, validation, and testing, which were 0.89, 0.77, and 0.91, respectively, the findings presented in the Fig. 4 prove a good connection. The quantity of datasets has a significant impact on the suggested model's reliability^36^. In this research 127 specimens for *TS* were gathered from the literature available to achieve better results.

## SHAP analysis

SHAP (SHapley Additive exPlanations) is a popular framework for explaining the output of machine learning models. It provides a way to understand the contribution of each feature to the model's predictions^37^. SHAP values are based on cooperative game theory and they give a clear understanding of how much influence an individual parameter has on targeted output. SHAP values are based on the concept of Shapley values, which originate from cooperative game theory. In cooperative game theory, Shapley values allocate a fair share of the total payoff to each player in a game based on their marginal contributions. In the context of machine learning, each feature in a dataset can be considered as a "player" in a cooperative game. The "payoff" is the model's prediction^38^.

SHAP values help explain why a particular prediction was made by breaking down the prediction into contributions from each feature.

Positive SHAP values indicate a feature's positive contribution to the prediction, while negative values indicate a negative contribution. The sum of SHAP values for all features equals the difference between the model's prediction for a specific instance and the expected (average) prediction. SHAP values can be calculated using various methods, depending on the model and the specific use case. Common methods include SHAP Kernel Explainer, Tree SHAP for tree-based models, Deep SHAP for neural networks, and more.

For tree-based models like decision trees or random forests, Tree SHAP is often used to efficiently compute SHAP values. SHAP values can be visualized using various techniques, such as SHAP summary plots, SHAP dependence plots, and force plots.

**Figure 5:**
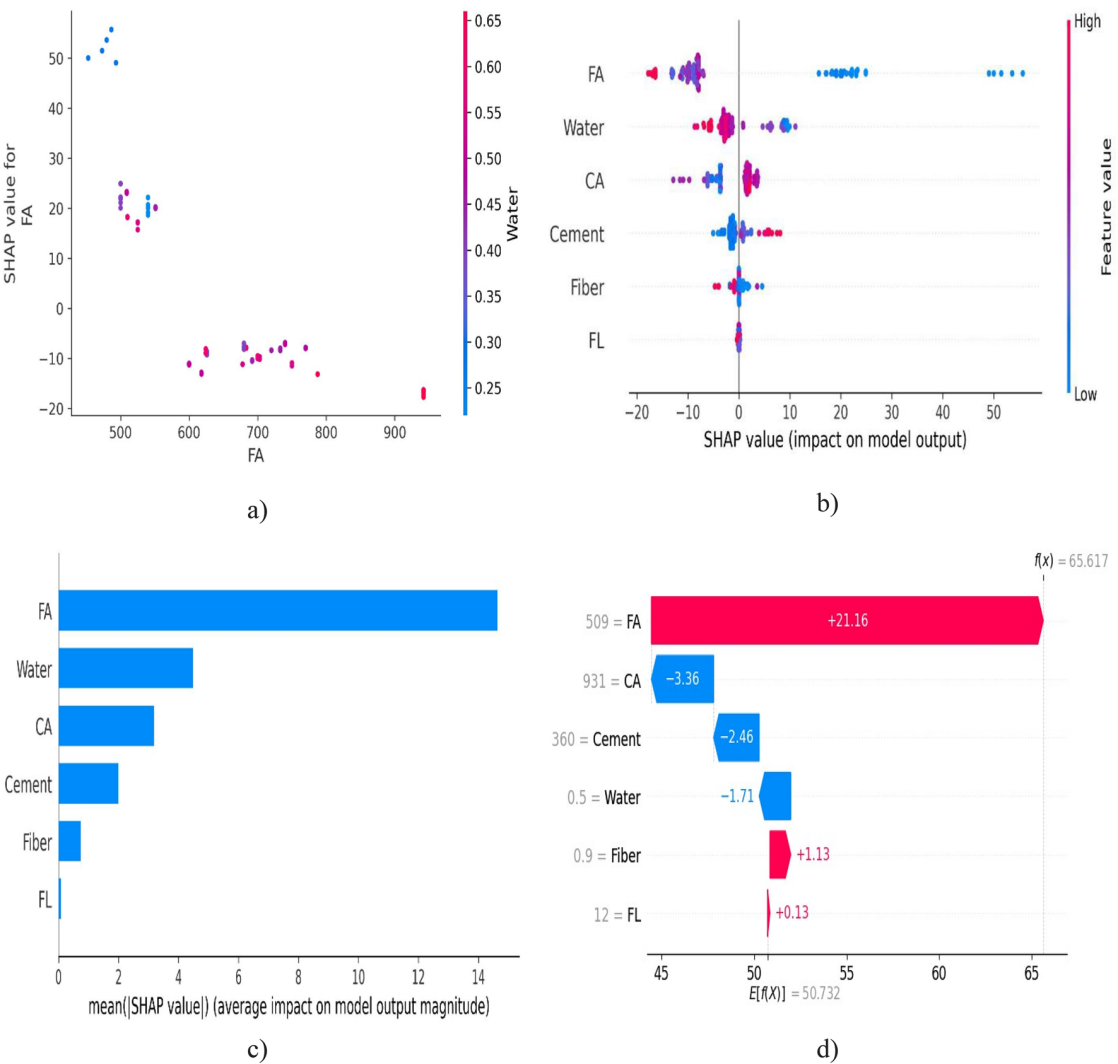
SHAP analysis for CS of BFRC.

**Figure 6:**
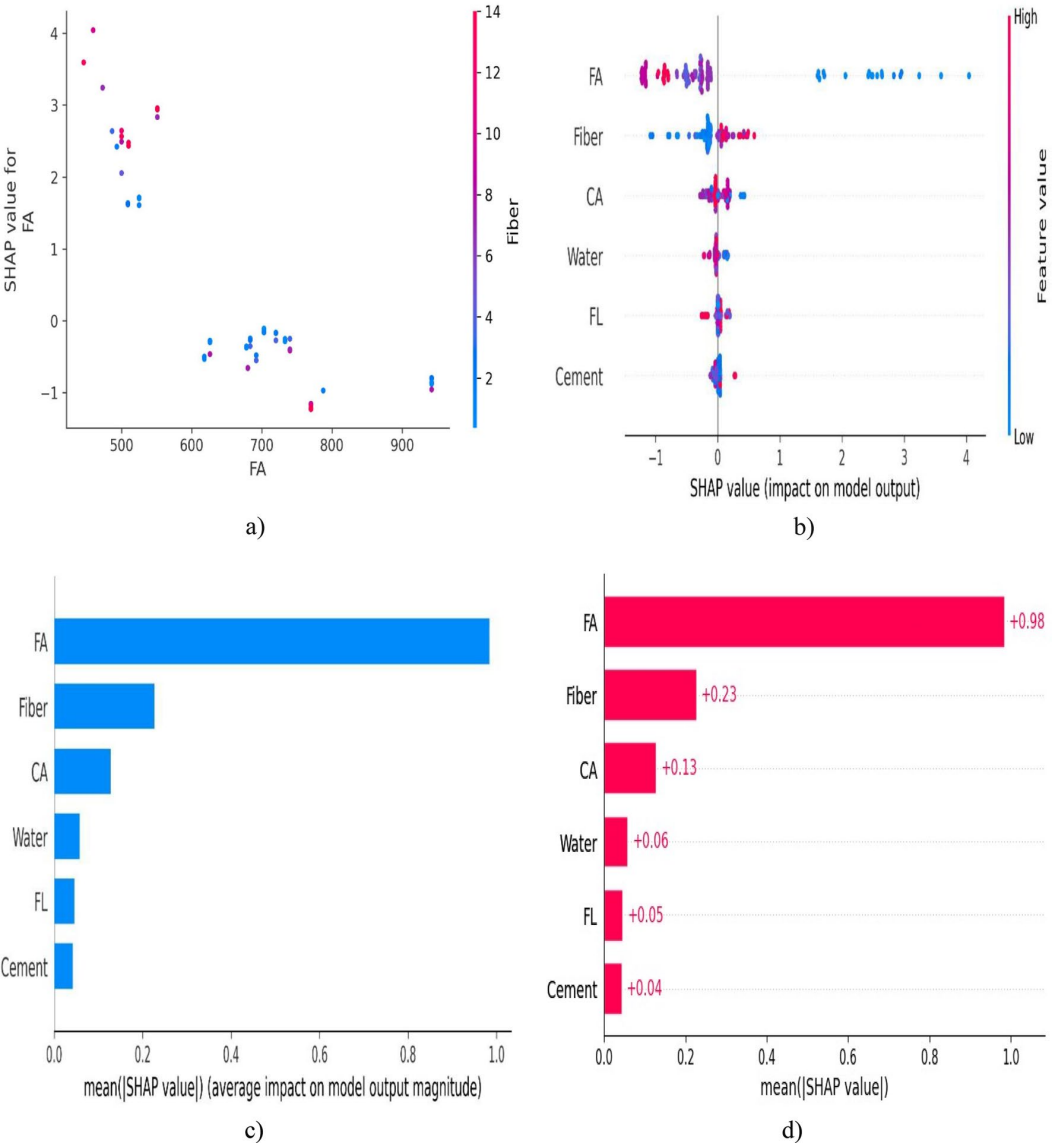
SHAP analysis for TS of BFRC.

Summary plots provide an overview of feature contributions across a dataset, while dependence plots show how a single feature's value impacts predictions. Force plots display the individual feature contributions for a single prediction. SHAP analysis is valuable for understanding black-box models, such as complex neural networks, and for building trust and accountability in machine learning systems. It's useful in various applications, including credit scoring, healthcare, image analysis, natural language processing, and more. You can perform SHAP analysis in Python using libraries like shap, which provides tools for calculating and visualizing SHAP values. In summary, SHAP analysis is a powerful technique for explaining the predictions of machine learning models. It helps users gain insights into the model's decision-making process and provides valuable interpretability and transparency, which are essential in many real-world applications. SHAP analysis for CS and TS can be clearly viewed in Figs. 5 and 6. In Fig. 5 FA is most influential parameter influencing compressive strength to BFRC. The other input parameters are listed in descending order as per their performance. For better understanding, one can easily observe the importance by higher positive value. Large positive value indicates more significance of these parameters.

As explained earlier, higher positive value of a parameter is an indicator of its dominancy while imparting strength to concrete. In Fig. 6 FA shows higher mean SHAP value. It shows that these values starting from higher range closer to 1 for FA ending on cement with lower value of 0304 show significance of parameters for TS of BFRC in descending order. It does not mean that parameters with lower value should be discarded because they are necessary for strong binding properties despite of the fact that they do not have much contribution in imparting strength.

### Performance assessment of machine learning models

**Figure 7:**
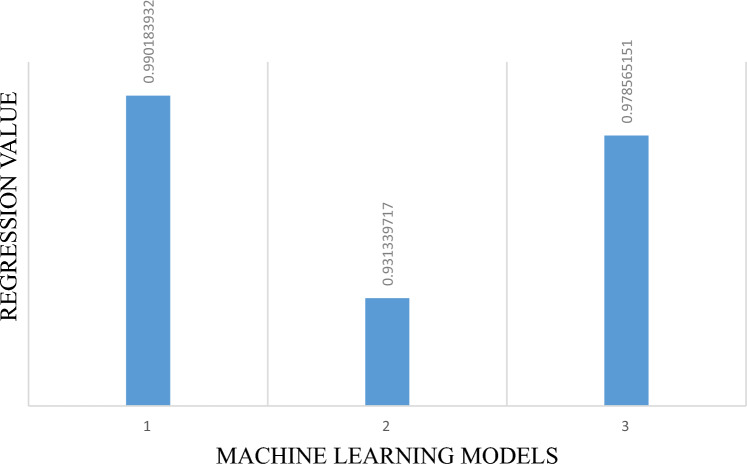
Comparison of performance indicator (R^2^) for three models.

The criteria used in this study to assess the model efficiency is to compare any performance indicator used in research for all Machine Learning models used. For this purpose Regression value (R^2^) is chosen in this study to validate the performance of GEP more clearly. In Fig. 7 bar 1 represents the R^2^ for GEP and 2, 3 for ANN and XG Boost. This figure clearly explains the importance of GEP chosen for this study because of its accuracy and robustness which can be seen by larger value of R^2^ for GEP.

### Relative study of GEP and machine learning models

To the knowledge of the researcher's understanding, no empirical methods have been created for calculating the CS of BFRC that would account for the crucial factor considered in this investigation. As a result, using comparable datasets, linear and non-linear regression along with ANN and XG boost models have also been proposed to calculate the CS of BFRC.

**Figure 8:**
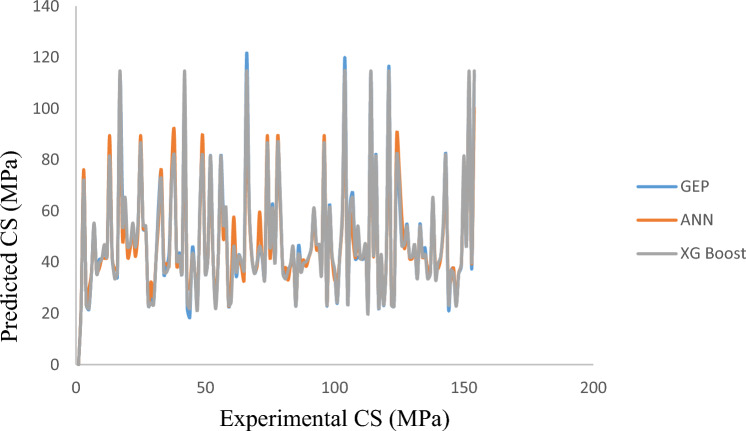
Formulating CS by GEP, ANN and XG Boost.

**Figure 9:**
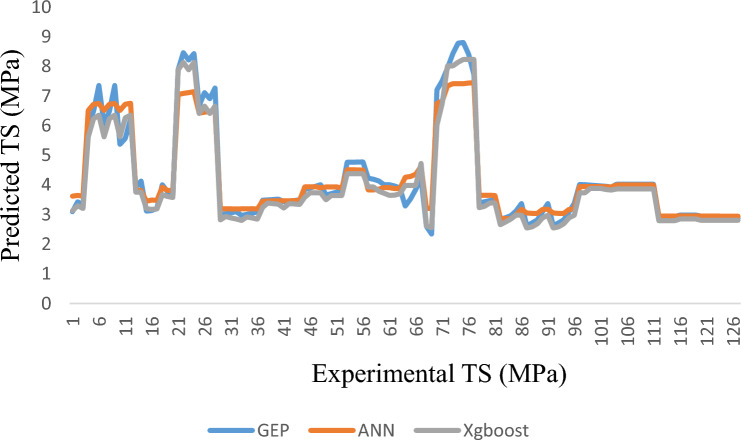
Formulating TS by GEP ANN XG Boost.

The comparison of the *CS and TS* findings suggested by the three models is shown in Figs. 8 and 9. For all three datasets, regression models follow the statistical parameters, RMSE and the GEP model, the least. In comparison to ANN and XG Boost, the RMSE training for the *CS* outcomes predicted by GEP is about 33.7% lower. Testing of the two models, which differ by 50%, shows that the GEP model performs better than ANN and XG Boost models during the testing phase. However, these three models are applicable for forecasting the CS and TS of BFRC as the targeted outputs in Figs. 8 and 9 show that data output values lie close to each other. This means that no widespread outliers were produced while processing these models.

Equations 18 and 19, respectively, provide the empirical equations to forecast *CS*.

18 $$CS_{{}} \left( {{\text{MPa}}} \right) \, = { 55}.{2}0 \, - { 32}.{7}0{\text{w}}/{\text{c }} + { 12}.{\text{61 BF}}\% \, - { 4}.{\text{89 BF}}/{\text{c }}{-}{ 3}.{\text{11 FA}}$$

19 $$CS_{{}} \left( {{\text{MPa}}} \right) \, = { 41}.{55 } - { 48}.{82}\left( {{\text{w}}/{\text{c}}} \right)^{{{3}.{98}}} + { 1}0.{\text{82BF}}\%^{{{2}.{79}}} {-}{ 1}.{7}0 \, \left( {{\text{BF}}/{\text{c}}} \right)^{{{1}.{95}}} {-}{ 1}.{\text{94BF}}^{{{1}.{47}}}$$

Equations 20 and 21, respectively, provide the empirical equations to determine *TS* relying on linear and non-linear regression analysis.

20 $$TS_{{}} \left( {{\text{MPa}}} \right) \, = { 9}.{61 } + \, 0.00{\text{4 C }} + \, 0.0{\text{8 F }}{-}{ 1}.{\text{26 w}}/{\text{c }}{-} \, 0.00{\text{232 CA }}{-} \, 0.00{\text{679FA }} + \, 0.0{\text{13FL}}$$

21 $$TS\left( {{\text{MPa}}} \right) \, = { 14}.{69} + {5}.{91}*{1}0^{{ - {8}}} \left( {\text{C}} \right)^{{{2}.{62}}} + {1}.{\text{64F}}^{{0.{19}}} {-}{7}.{21 }\left( {{\text{w}}/{\text{c}}} \right)^{{0.{1}0{9}}} {-}{3}.{2}*{1}0^{{ - {6}}} {\text{CA}}^{{{1}.{85}}} {-} \, 0.0{\text{276 FA}}^{{0.{8}0{8}}} + 0.0{\text{898FL}}^{{0.{547}}}$$

Golbraikh et al. (2002) suggested that one of slopes of regression line (*k or k’*) should be close to 1 if it passes through the origin. As can be seen, regression line of *CS* slope is 0.99 and that of *TS* is 0.89. This suggests more precision and correlation. It should also be near to 1 for the squared correlation coefficient (through the origin) between experimental and predicted values or for the coefficient between predicted and experimental values. This is according to several scholars. Table 6 shows that the model complies with the requirements for external verification, demonstrating that the GEP models are highly valid, demonstrate the ability to predict, and go beyond simple correlations between input and output characteristics.

**Table 6:** Statistical parameters of GEP model for external verification.

Sr. no	Equation	Condition	Model	Model
			*CS*	*TS*
1	${\text{k}} = { }\frac{{\mathop \sum \nolimits_{i = 1}^{n} \left( {e_{i} \times m_{i} } \right)}}{{e_{i}^{2} }}$	0.85 < k < 1.15	0.99	0.89
2	${\text{k}}^{\prime} = { }\frac{{\mathop \sum \nolimits_{i = 1}^{n} \left( {e_{i} \times m_{i} } \right)}}{{m_{i}^{2} }}$	0.85 < k’ < 1.15	1.00	0.82
3	$R_{o}^{2} = 1 - { }\frac{{\mathop \sum \nolimits_{i = 1}^{n} \left( {m_{i} - e_{i}^{o} } \right)\left( {m_{i} - e_{i}^{o} } \right)}}{{\mathop \sum \nolimits_{i = 1}^{n} \left( {m_{i} - m_{i}^{o} } \right)\left( {m_{i} - m_{i}^{o} } \right)}}, e_{i}^{o} = k \times m_{i}$	$R_{o}^{2} \cong 1$	0.98	0.96
4	$R_{o}^{^{\prime}2} = 1 - { }\frac{{\mathop \sum \nolimits_{i = 1}^{n} \left( {e_{i} - m_{i}^{o} } \right)\left( {e_{i} - m_{i}^{o} } \right)}}{{\mathop \sum \nolimits_{i = 1}^{n} \left( {e_{i} - e} \right)\left( {e_{i} - e_{i}^{o} } \right)}}, m_{i}^{o} = k^{\prime} \times e_{i}$	$R_{o}^{^{\prime}2} \cong 1$	0.99	0.98

The coefficient between predicted and experimental values, or the squared correlation coefficient (via the origin) between experimental and predicted values, should also be near to 1, according to various scholars. It can be observed that GEP model not only correlates the input and output parameters but also is found efficient in prediction, validation, and verification of the data. Soft computing techniques^39–41^, deep learning algorithms^42–44^ and machine learning^45–49^ can be utilized for further analysis. Moreover using artificial neural network^50,51^, support vector machines^52,53^; random forest^54,55^, deep learning neural network^56,57^, neuro fuzzy^58,59^ and extreme learning^60,61^, support vector machines^62–64^ and hybrid machine learning model of genetic algorithm^65–67^ can be utilized to predict the response using the existing experimental data. It will save the cost and human effort as well and open new directions for future research. Zhou et al.^68^ and Wu et al.^69^ conducted separate studies on the moisture diffusion coefficient of concrete under various conditions and the three-dimensional simulation of seismic wave propagation, taking into account source-path-site effects. Xu et al.^70^ and He et al.^71^ investigated mine water inflow from roof sandstone aquifers using upscaling techniques and the use of N-doped graphene quantum dots to enhance the chloride binding of cement, respectively. Zhan et al.^72^ and Zhou et al.^73^ performed data-worth analysis for identifying and characterizing heterogeneous subsurface structures and developing high-strength geopolymer based on BH-1 lunar soil simulant, respectively. Tian et al.^74^ and Ren et al.^75^ studied the collapse resistance of steel frame structures in column-loss scenarios and developed a damage model for porous rock suitable for different stress paths, respectively. Cheng et al.^76^ and Yu et al.^77^ investigated the effects of methane and oxygen on heat and mass transfer in reactive porous media and the stress relaxation behavior of marble under cyclic weak disturbance and confining pressures, respectively. Xu et al.^78^, Ren et al.^79^, and Yao et al.^80^ have recently examined the properties of source rocks and the genetic origins of natural gas. They have also investigated the damage caused by compaction and cracking, as well as the combined disturbance-induced damage to rocks.

## Conclusions

This research depicted the application of gene expression programming (GEP) strategy to predict CS and TS of BFRC. The proposed model is empirical and relies on widely dispersed catalogue gathered from different experimental datasets studied in literature.

The results obtained from the predicted model validate the experimental findings. The analysis of parameters depict that the suggested model agrees with the contribution of input parameters to suggest the accuracy in the trend of CS and TS of BFRC as can be seen in SHAP Analysis and in Figs. 8 and 9.

The assessment and comparing of fitness functions (β and OBF) and statistical parameters (RMSE and R) for all three sets (training, validation, and testing), revealed the precision of the suggested models in Table 6.

Additionally, the model clearly satisfies various criteria considered for external validation. When derived GEP and regression models are compared, it becomes clear that GEP models outperform ANN and XG Boost models in terms of generalization and prediction, making them ideal for implementing in designing of BFRC by observing R^2^ value of 0.99 in Fig. 7.

It is recommended to perform dredged assessment first before using BF as reinforcement in concrete. It is better to carefully consider the optimum content of BF to achieve desired CS and TS of BFRC in Figs. 1 and 2.

Instead of dumping basalt fibers as trash, the empirical models can supply a precise and powerful foundation for enhancing their use in construction projects. This may help create high-performance concrete, which will be more useful in the construction sector. Further study on BFRC can be done for other parameters like Elasticity and torsional effects by using GEP which will aid in more deep understanding of properties of BFRC^1,2^. For future studies on BFRC, several other machine learning techniques like^3–5^ can be implemented.

## Data Availability

The data will made available on a reasonable request to the corresponding author.
